# Breast Implants or Lipofilling in Augmentation Mammoplasty? A Randomized, Open-Label, Controlled Trial

**DOI:** 10.1007/s00266-025-05204-0

**Published:** 2025-09-04

**Authors:** Pietro Gentile

**Affiliations:** Department of Surgical Science, Medical School, Plastic and Reconstructive Surgery, Tor Vergata” University, 00133 Rome, Italy

**Keywords:** Breast hypoplasia, Breast lipofilling, Breast implants, Fat and implants in comparison, Plastic surgery, Stromal vascular fraction

## Abstract

**Background:**

The author presents his own experience using breast implants (BIs) or fat grafting, commonly called lipofilling (LPF), to correct breast hypoplasia.

**Objectives:**

Compare the aesthetic results obtained in a study group (SG) using BIs in breast hypoplasia correction with those of a control group (CG) treated with LPF, analyzing the influence of breast and chest deformities (tuberous breast, breast volume differences/asymmetries, nipple–areola complex asymmetry, pectus excavatum, and carinatum) in the outcomes.

**Methods:**

A randomized, open-label controlled study was performed. A total of 95 patients affected by breast hypoplasia (SG) were treated with BI, comparing results with the CG (n = 90) treated with LPF. The pre-operative analysis was conducted through anamnesis (considering also the patient’s expectations), clinical and photographic assessment, and an instrumental evaluation based on magnetic resonance imaging, mammography, and ultrasound. Post-operative follow-up occurred at 1, 2, 4 weeks, 3, 6, 12 months, and then annually until the fourth year.

**Results:**

87,5% (n = 83) of SG patients treated with BI showed excellent aesthetic outcomes after 12 months compared with the CG patients treated with LPF, who showed the same results in 70% (n = 63) of cases. Breast augmentation maintenance in the SG was significantly higher than in the CG (*p *< .0001). However, more natural results were reported in the CG than in the SG (*p *< .0001).

**Conclusions:**

BI and LPF were safe and effective in this controlled trial. CG's patients displayed more natural results, obtaining a better pectus excavatum correction, while SG’s patients showed more evident and lasting results.

**Level of Evidence I:**

This journal requires that authors assign a level of evidence to each article. For a full description of these Evidence-Based Medicine ratings, please refer to the Table of Contents or the online Instructions to Authors www.springer.com/00266.

**Supplementary Information:**

The online version contains supplementary material available at 10.1007/s00266-025-05204-0.

## Introduction

Augmentation mammoplasty is a surgical procedure that requires a thorough understanding of the relationships between the chest (including muscles, glands, fat, soft tissues, dermis, and skin) and breast (shape, volume, nipple position). The thickness of the breast tissue plays a key role in determining the procedure approach, particularly the anatomical plane in which the implant will be placed (sub-glandular, submuscular, or dual plane). Therefore, it is essential to understand how to intervene in these tissues to achieve the desired results using the breast implant (BI) [1].

Over the past twenty-five years, surgical procedures for augmentation mammoplasty have evolved, shifting from an exclusive reliance on BI to less invasive mammoplasty techniques that do not involve BI and are based solely on autologous fat graft injection, commonly called “lipofilling” (LPF) [2]. This strategy for breast augmentation involves “*minimal manipulation*” of fat tissue through several methods like washing, decantation, centrifugation, filtration, or enzymatic digestion using human collagenases [2]. The goal is to obtain purified fat to inject during the LPF procedure containing a higher concentration of adipose-derived mesenchymal stem cells (AD-MSCs), which improve the fat graft retention. AD-MSCs are found in the stromal vascular fraction (SVF) of subcutaneous tissue, which contains a heterogeneous set of mesenchymal cells [3]. SVF cells (SVFs) can be isolated and/or concentrated further through enzymatic digestion or by combining filtration and centrifugation as a mechanical digestion [4].

A limitation of LPF is fat resorption. Recent studies on augmentation mammoplasty procedures using LPF have shown that when LPF is enriched with AD-MSCs or contains a high concentration of AD-MSCs (after minimal manipulation procedures to purify LPF), 58% of the fat volume is maintained after three years. In contrast, not purified LPF with a poor amount of AD-MSCs resulted in only 29% fat volume retention. [2]

On the other hand, breast implants (BIs) have been associated with potential side effects, including implant displacement, deformities, rejection, wrinkling, and rippling [1]. More recently, there has been an association with anaplastic large-cell lymphoma [1]. These issues have prompted several plastic surgeons to develop less invasive strategies for augmentation mammoplasty. Additionally, improper resection of the pectoralis major muscle can lead to severe complications, such as the “jumping breast” phenomenon [1, 5]. An accurate evaluation of both breast soft tissue (primarily muscle, gland, and fat) and the patient**’**s expectations plays a crucial role in the decision between using BI and LPF [1, 2, 4, 5]. Furthermore, when considering LPF, it is important to assess the amount of fat available in the flanks, abdomen, and thighs. Very thin patients are generally not considered suitable candidates for this procedure.

For these reasons, this trial led to a comparison of the cosmetic outcomes of breast hypoplasia correction using BIs with those achieved through LPF. The study also analyzed how breast and chest deformities (such as tuberous breasts, volume, nipple–areola complex [NAC] asymmetry, pectus excavatum and carinatum, and low body mass index [BMI]) influence the cosmetic outcome. The advantages and disadvantages of each technique were discussed. Study limitations and strengths were also considered.

## Methods

### Study Overview

A randomized, open-label, controlled study meeting the criteria for evidence-based medicine (EBM) level 1 was conducted following the principles outlined in the Declaration of Helsinki and internationally accepted ethical guidelines for clinical research [6]. A quality assessment was conducted using the consolidated standards of reporting trials (CONSORT) guidelines (http://www.consort- statement.org) (Scheme 1) and strengthening the reporting of observational studies in epidemiology (STROBE) checklist [7] (Supplemental Material 1). All patients received detailed oral and written information about the study, including the associated risks, benefits, and alternative therapies, and signed an informed consent form before any study procedures. The study protocol adhered to European regulations (1394/2007 EC) and EMA/CAT recommendations (20 June 2014 EMA/CAT/600280/2010 Rev 1). The investigation protocol was developed in agreement with an associate professor contract #13489/2021 between the author and the University of Tor Vergata, Rome, Italy, and as a part of a research project approved by the Surgical Science Department with number: E83C22001960005.

**Sch 1:**
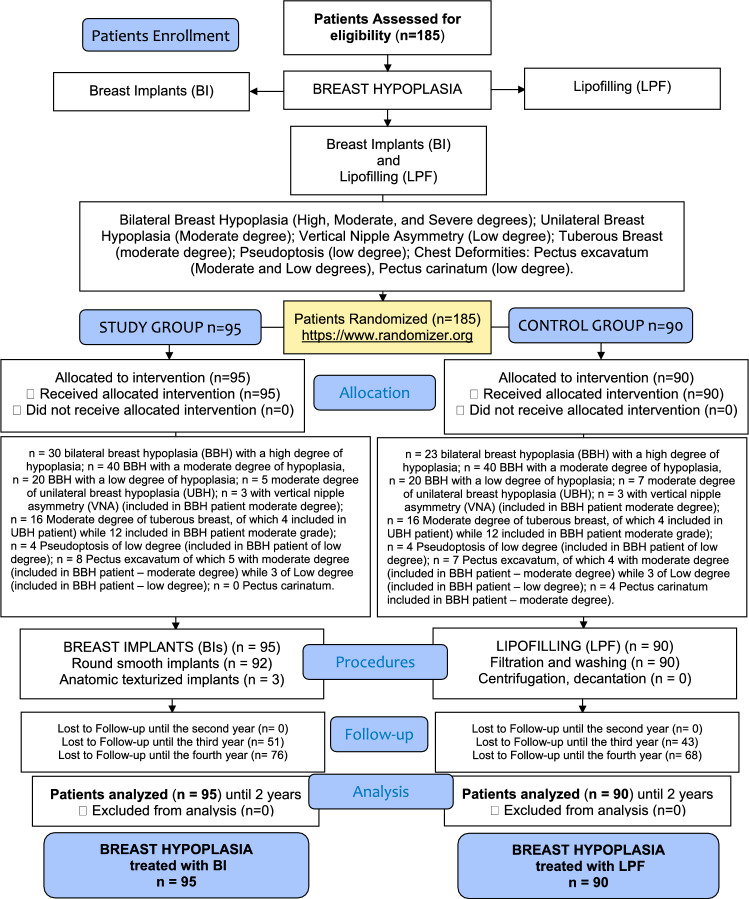
CONSORT (Consolidated Standards of Reporting Trials) flow diagram on patients’ enrollment, including soft tissue defects and treatment assessment.

### Patients Recruitment

**Fig. 1:**
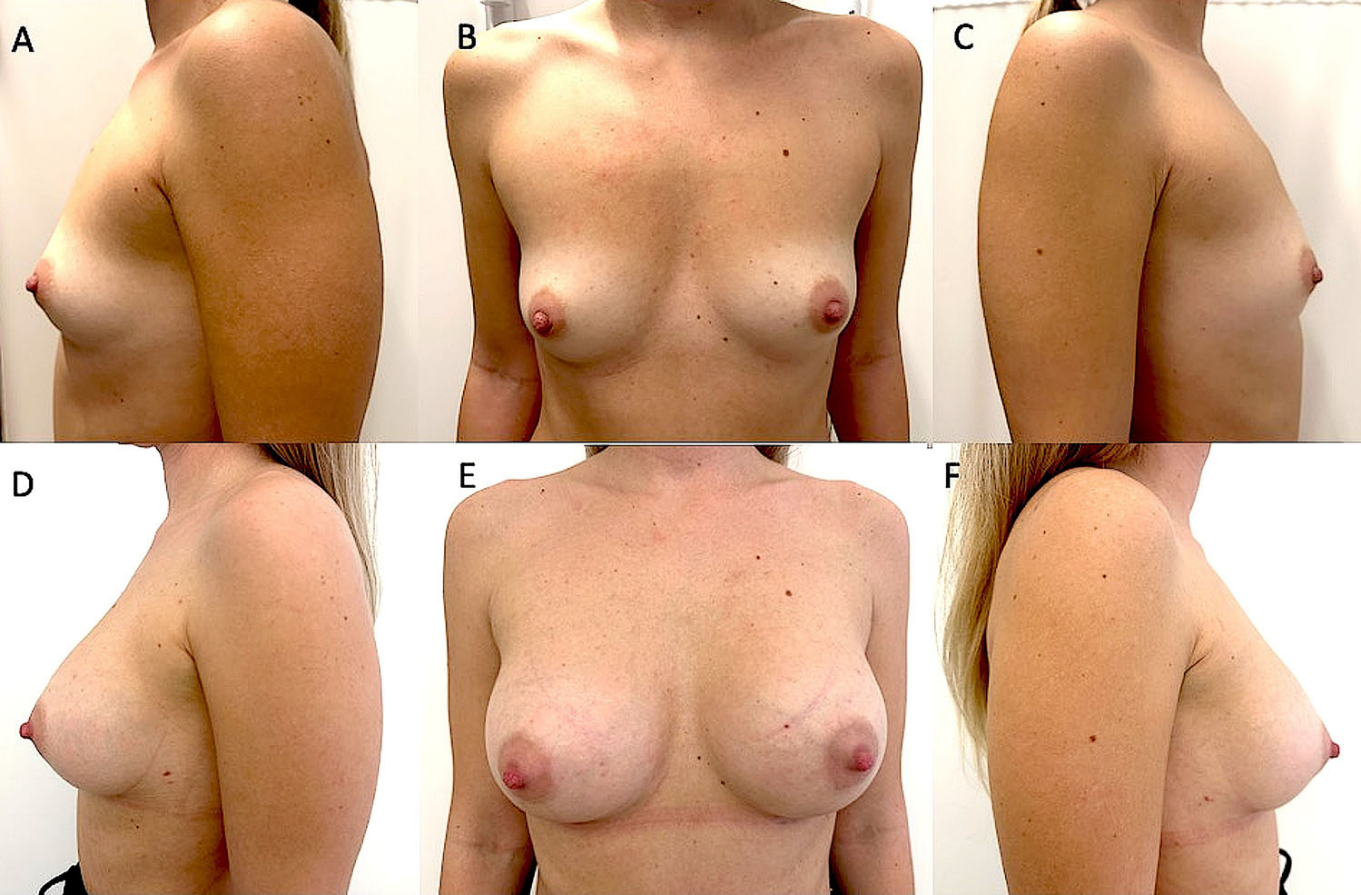
A 29-year-old female affected by a moderate degree of bilateral breast hypoplasia (BBH) was treated with breast silicone implants (BIs). **A**) Pre-operative projection in ¾ left view. **B**) Pre-operative projection in frontal view. **C**) Pre-operative projection in ¾ right view. **D**) Post-operative projection after 1 year (T6) in ¾ left view. Breast silicone implants of 300cc, round and smooth matte (Perle–GC Aesthetics) were applied bilaterally under the glands (over the muscle). **E**) Post-operative projection after 1 year in frontal view, evidencing the significant breast improvement after one surgical procedure. **F**) Post-operative projection after 1 year in ¾ right view.

**Fig. 2:**
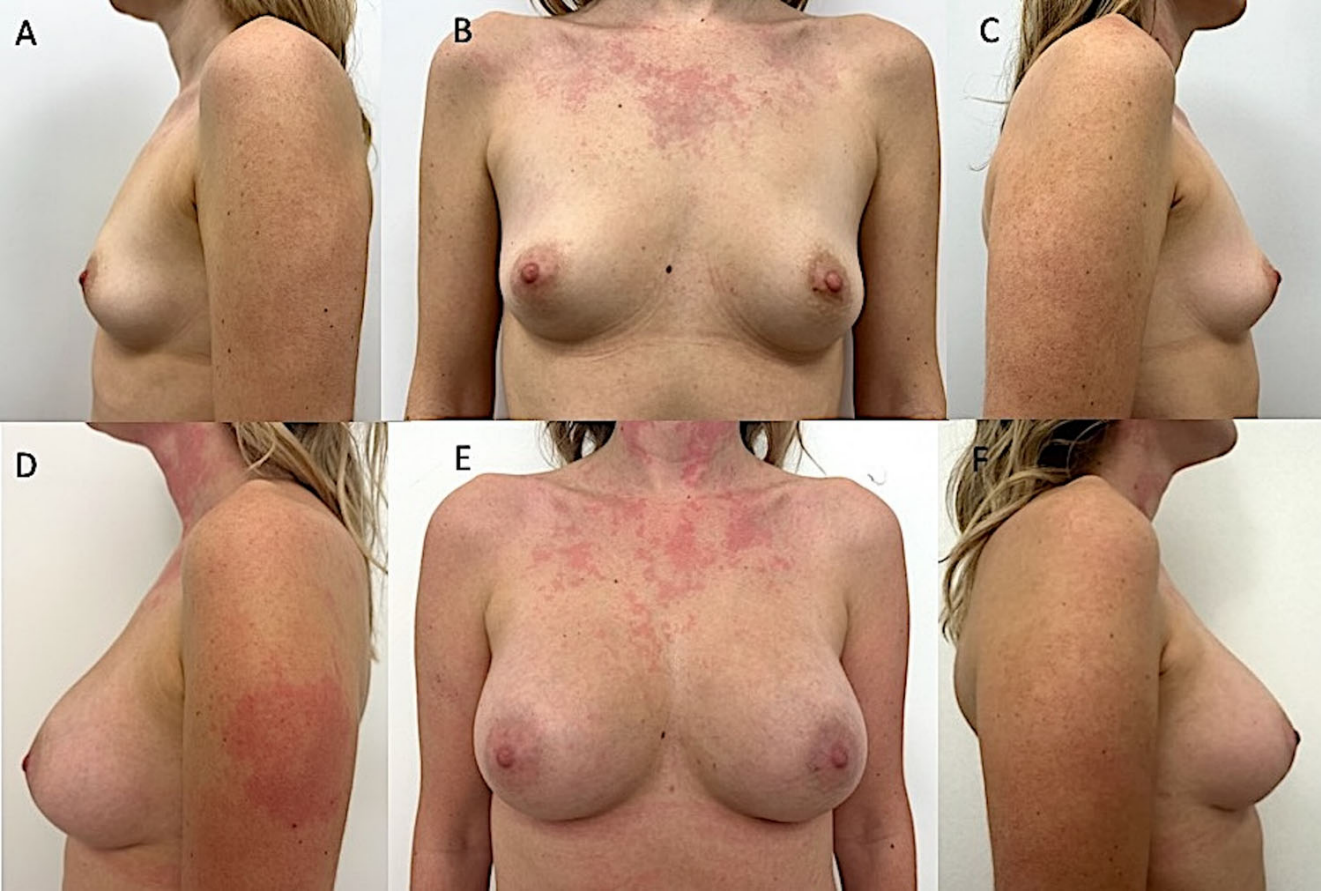
A 30-year-old female affected by a moderate degree of bilateral breast hypoplasia (BBH) was treated with autologous lipofilling (LPF). **A**) Pre-operative projection in ¾ left view. **B**) Pre-operative projection in frontal view. **C**) Pre-operative projection in ¾ right view. **D**) Post-operative projection in ¾ right view after two LPFs. **E**) Post-operative projection in frontal view 12 months after the second LPF. During the first LPF, 180ml for each breast was injected. One year later, a second LPF was performed, injecting 210ml of fat for each breast. An increase of 72.8mm in the three-dimensional volume after 1 year (T6) was observed, evidencing a cosmetic result comparable with that obtained by BIs. **F**) Post-operative projection in ¾ right view and two LPFs evidencing the naturalness of the outcome.

Over 4 years (2021–2025), 185 patients affected by several degrees of breast hypoplasia were randomly assigned to two groups; 95 patients (study group, SG) were treated with augmentation mammoplasty using BIs. The SG consisted of 40 patients with moderate-degree bilateral breast hypoplasia (BBH) (Fig. 1A-C), 30 patients with high-degree BBH, 20 patients with low-degree BBH, and 5 patients with unilateral breast hypoplasia (UBH). The SG comprised 95 females aged 18–61 years (mean age 39.5). Of these, 74 (75.8%) were premenopausal. To assess the long-term follow-up of cosmetic results, the author compared the outcomes with a control group (CG) of 90 patients treated with LPF. The CG included 90 females aged 18–58 years (mean age 38), all diagnosed with breast hypoplasia (23 patients with high-degree BBH, 40 with moderate-degree BBH [Fig. 2A-C], 20 with low-degree BBH, and 7 with UBH). In the CG, 69 (77%) were premenopausal. All patients in both SG and CG were females and underwent a full pre-operative screening, including a detailed anamnesis (also evaluating the patients**’** expectations in terms of breast volume and shape), clinical evaluation, photographic analysis, and instrumental assessments using magnetic resonance imaging (MRI), ultrasound (US), and mammography (MG).

### Inclusion and Exclusion Criteria

Inclusion criteria were as follows: age 18–70 years, a history of high, moderate, or low-degree breast hypoplasia (bilateral and/or unilateral). Additional inclusion criteria for both groups included a BMI between 20 and 35 kg/m^2^. For the CG, additional criteria required sufficient fat reserves in the abdomen, thighs, flanks, and inner knees (the sites of fat harvest).

Exclusion criteria were divided into local and systemic categories. Systemic exclusion criteria included anti-aggregating therapy, bone marrow aplasia, uncompensated diabetes, sepsis, and cancer. Local exclusion criteria included breast ptosis, reconstruction with expanders, first-stage LPF followed by BI, reconstruction with BI followed by LPF, cancer-related tissue loss, and uncontrolled comorbidities. Previous breast surgery, tobacco use, outcomes of breastfeeding, prior history of COVID-19, and genetic disorders were not considered exclusion criteria.

### Clinical Appraisal

A multidisciplinary team discussed and decided upon all therapeutic strategies, including two plastic surgeons, a radiologist, and a psychologist. Patient information (demographic data, age, BMI, breast defects, surgical management, surgical complications, and clinical appraisal) is, respectively, described in Scheme 1, a CONSORT flow diagram, and Table 1. All the participants were identified based on similar breast deformities, aiming to create two homogeneous groups. Patients were analyzed at T1—1 week, T2—2 weeks, T3—4 weeks, T4—3 months, T5—6 months, T6—12 months, T7—24 months, T8—36 months, T9—48 months through clinical and photographic evaluations, while at T6, T7, T8, T9, through MRI and US. Deformities and asymmetries were documented through clinical examinations, pictures, and instrumental tests. Preoperative and post-operative imaging included standard front, left/right lateral, and left/right oblique views.

**Table 1:** Patients’ data and clinical assessment

	BI	LPF
	Study group	Control group
Number of patients, no°	95	90
Race	Caucasian	Caucasian
Age at surgery, yr	39,5 (min 18, max 61)	38,0 (min 18, max 58)
BMI at surgery, kg/m2	27,5 (min 20, max 35)	27,5 (min 20, max 35)
Bilateral breast hypoplasia (BBH)	40 (moderate) (42,2%),	40 (moderate) (44,5%),
	30 (high) (31,6%),	23 (high) (25,6%),
	20 (low) (21,1%)	20 (low) (22,3%)
Unilateral breast hypoplasia	5 (moderate) (5,3%)	7 (moderate) (7,8)
Pre-menopausal	74 (75,8%)	69 (77%)
Only one session	86 (90%)	50 (56%)
Mean BI or LPF transfer volume for each breast during the first procedure	235mL (range 150-320mL)	180mL (range 80-280mL)
Second session	9 (9,5%)	40 (44,5%)
	mastopexy n = 1; implant replaced n = 2; surgery for capsular contracture n = 6)	
Mean BI or LPF transfer volume for each breast during the second treatment	285 ml (range 150–420ml)	210 mL (range 130–290 mL)
Implant’s shape for each breast	92 Round (96,9%)	–
	3 Anatomical (3,1%)	
Implant’s surface for each breast	92 Smooth matte (96,9%)	–
	3 Texturized (3,1%)	
Implant’s profile for each breast	10 High (10,6%)	–
	80 Moderate (84,3%)	
	5 Low (5,3%)	
Fat and implant’s positioning plane for each breast	80 Sub-glandular (84,2%)	90 Retro-glandular, Intra-glandular, Sub-cutaneous (100%)
	5 Sub-muscular 5,3%	
	10,6% 10 Dual-plane (10,6%)	
Incisions for LPF or BI positioning	24 periareolar incision (25,3%)	90 Superior and inferior lateral and internal quadrants, the peri-areolar region, and the superior and inferior poles (100%)
	71 inframammary fold incision (73,8%)	
Drains	100% all patients	N/A
Volume maintenance percentage (1 year later)	100% (All patients)	68.8% ± 5% (All patients)
Volume maintenance percentage (3 years later)	100% (48 patients) (46,4%)	53% ± 5% (52 patients) (57,8%)
Cyst formation and calcification	0	0
Fat necrosis	0	0
Breast ptosis	1 (1.1%)	0
Inadequate final volume	2 (2.2%)	0
Capsular contracture	6 (6,4%)	0
Double bubble	0	–
Wrinkling	2 (2,2%)	–
Rippling	0	–
Intra/Extracapsular ruptures	0	–
Implants/fat displacement	0	0

### Instrumental Appraisal

A multimodal imaging approach based on the combined use of MRI and US was adopted. In detail, MRI and US were used to analyze the BI conditions (shell integrity, intra-extra-capsular rupture, displacement, and capsular contracture), breast volume, and to perform LPF assessment (detecting macro- and micro-calcifications, oil cysts, fat necrosis and analyzing LPF volume maintenance) as reported in Table 1. A 1.5 Tesla scanner (Hitachi, MS, Echelon Oval, Tokyo, Japan) was used, with 3-mm-thick slices. OsiriX software (Pixmeo, CA, USA), 32 bits, free version, was employed to calculate breast volume. Each examination included two volume calculations, and the average of these was taken as the final breast volume. Based on the MRI scans, volumetric assessments of the LPF distribution in the breasts were also conducted, considering the anterior axillary line, anterior margin of the pectoral muscle, mid-sternal line, skin, and nipple as the boundaries. These were evaluated using three-dimensional reconstruction on a separate workstation (ADW 4.0; GE Medical Systems, Milwaukee, Wis.).

### Allocation Sequence

The patient allocation sequence for both the SG and CG groups, based on the aforementioned inclusion and exclusion criteria, was generated using an online randomization tool (https://www.randomizer.org) and was kept concealed by someone not involved in the trial management. The treatment allocation was known to the participants, study personnel, and outcome assessors.

### Quality Checks

Quality checks were performed on all treated patients based on the following criteria:

- Surveys related to the patient**’**s satisfaction level on breast size, breast shape, breast lift, breast and NAC symmetry, scar quality, sexual well-being, availability to perform the procedure again, suggesting the treatment to friends, and sufficient information on the randomized trial, risks, and side effects (range vote from 4 [very dissatisfied] to 9 [Excellent] (Supplemental Material 2)
- Clinical evaluation based on the physician**’**s global assessment score (excellent [9], good [8], discreet [7], enough [6], poor [5], and inadequate [4]);
- Clinical evaluation based on the patient**’**s global assessment score (excellent [9], extremely satisfied [8], satisfied [7], neutral [6], dissatisfied [5], and very dissatisfied [4]);
- Visual Analog Scale (VAS) (range 1–10);
- Analysis of additional factors/variables, such as volume asymmetries, breast and chest deformities, NAC asymmetries, pseudoptosis, pectus excavatum and carinatum, low BMI, and very thin patients;
- Adverse effects signaling.

### Augmentation Mammoplasty with BIs: Surgical Planning in SG Patients

All SG patients treated received round implants, except for three, who requested anatomical implants. The profile (low, moderate, or high) and surface (smooth matte or textured) of the implants were chosen based on preoperative breast volume, skin quality, and patient preferences or expectations. If the patient had nipple asymmetry (vertical or horizontal NAC malposition) or tuberous breasts with hypoplasia, a peri-areolar incision was used. In all other cases, an inframammary incision was chosen (Table 1). Initially, smooth matte implants were preferred for submuscular and dual-plane pockets, while textured implants were preferred for sub-glandular pockets. Currently, smooth matte implants (Perle®) are used for all the approaches. All the BIs adopted were GC Aesthetics® (GC Aesthetics® Suite 601, Q House, Furze Road, Sandyford, Dublin 18, Ireland, company number: IE450181, www.gcaesthetics.com). The size of the definitive implants ranged from 150 to 320 cc during the first treatment. The choice between anatomical and round BIs or insertion planes was based on the available tissue, with “custom-made” methods used in all cases. Drains were applied in all SG patients. Vertical and radial cutting of the inferior gland was performed if necessary to expand the lower breast segment or in cases of tuberous breasts.

### Augmentation Mammoplasty with Autologous LPF: Surgical Fat Injection in CG Patients

LPF was injected with a “custom-made approach” based on the breast defect, tissues available, and patient expectations using the “Gentle Technique [8]”, which involves slow, controlled injections of fat in linear deposits across the supra-fascial, retro-glandular, and intra-glandular spaces (never into the pectoralis major muscle) through 1.5-mm cannulas [8, 9]. The LPF was injected using 10-ml and 2.5-ml Luer-Lok syringes, respectively, for deep and superficial planes into seven regions: the superior and inferior lateral and internal quadrants, the peri-areolar region, and the superior and inferior poles. The area for LPF injection was determined for each patient based on MRI scans and clinical assessment. In total, 80–280 mL (average 180 mL) of LPF was injected per breast during the first treatment, with a total range of 160–560 mL per patient.

### Augmentation Mammoplasty with Autologous LPF: Fat Processing

The LPF was prepared using “minimal manipulation” procedures based on filtration and washing procedures; 30 patients were treated with washing and filtration using Beauty-Stem™ medical device (BPB Medica©–BIOPSYBELL S.R.L. Via Aldo Manuzio 24, 41037 MIRANDOLA (MO), Italy, (https://www.biopsybell.com/%20products/aesthetic/). Up to 400 ml of fat was collected via a 3-mm-diameter cannula connected with a 50-cc vacuum Luer-Lok syringe and purified using a continuous washing procedure with saline, which filtered the fat tissue by continuously moving the processing spatula on the filter; 30 patients were treated with filtration and washing procedure using Euromi NLF system (Zoning Industriel des Plenesses 11 rue des Nouvelles Technologies 4821 Andrimont (BE)) (https://www.euromi.com/en/catp/lipofilling-nlf-system/). In this case, a 1000-mL closed inline filtration system was used, and fat (800 mL average in all patients—range 400/1200 mL) was collected and purified directly using a standard suction connected directly to the canister, permitting to filtering non-viable cells during harvest, saving valuable surgical time; 30 patients were treated with a filtration and washing procedure using Tissu-Trans Filtron—Class IIa product (Summit Medical, LLC, 815 Vikings Parkway, Suite 100, Saint Paul, MN 55121 USA, https://shippertmedical.com/products/tissutrans- filtron-250). Also in this case, a 1000-mL closed inline filtration system was used, and fat (800 mL average in all patients—range 400/1200 mL) was collected and purified directly without any procedure of centrifugation, using a standard suction connected directly to the canister.

### Fat’s Donor Site and Anesthesia

The donor site (abdomen, flanks, thighs, or inner knees) is infiltrated with a cold solution containing 1 mL of adrenaline per 500 mL of saline solution to minimize bleeding during the procedure. An inverse relationship has been observed between the amount of blood in the harvested fat and the viability of the adipocytes. [2, 4, 8] Local anesthesia is not used; the procedure is performed under sedation and general anesthesia. Fat tissue is harvested after 6 minutes using a 3-mm-diameter cannula and a 60-cc Toomey syringe.

### LPF Amount Evaluation to Inject Into Each Breast

Each breast was treated as a “cone” geometric shape. For this reason, the geometric formula volume = π × r^2^ × h / 3 (base area × height, divided by 3) was used to estimate the initial volume of each breast.^2^ The patient**’**s needs were aligned with the calculated initial breast volume and the amount of fat available. The optimal fat volume for injection was determined using the formula V = π × r^2^ × h / 3, with the same volume of fat (in milliliters) injected as the initial breast volume in cm^3^. [2]

### Complicating Factors

Breast deformities (e.g., high-degree BBH, UBH, tuberous breast, pseudoptosis), chest deformities (e.g., pectus excavatum or carinatum), abnormalities in NAC placement, and very thin patients were “complicating factors” influencing negatively the surgical outcomes producing non-optimal results, here defined “suboptimal results”. To determine how the results were influenced, the deformities correlation coefficient (DCC) was also computed. The incidence of each deformity was analyzed, and the number of preoperative deformities for each patient was determined (Table 2). The relationship between these deformities and suboptimal surgical results was evaluated. The analysis of the patient**’**s deformities and clinical features has led to the identification of several potential contributing variables to the surgical outcome**’**s downgrading as well as the occurrence of each of these deformities. For these reasons, complicating factors were distinguished into major complicating factors (M-CFs) and minor complicating factors (m-CFs). M-CFs were those that, when present alone, produced a “suboptimal result,” whereas m-CFs did so only when present in combination with two (Table 3).

**Table 2:** Breast and chest deformities

Deformities	Grade	BI Group (90)	LPF Group (90)
Bilateral breast hypoplasia (BBH)	High	30 (31,6%)	23 (25,6%)
	Moderate	40 (42,2%)	40 (44,5%)
	Low	20 (21,1%)	20 (22,3%)
Unilateral breast hypoplasia (UBH)	Moderate	5 (5,3%)	7 (7,8%)
Vertical NAC asymmetry	Low	3 (included in BBH patient moderate grade) (3,2%)	3 (included in BBH patient moderate grade) (3,4%)
Horizontal NAC asymmetry	Low	5 (included in BBH patient moderate grade) (5,3%)	4 (included in BBH patient moderate grade) (4,5%)
Areolar prolapse	Low	2 (included in BBH patient moderate grade) (2,2%)	2 (included in BBH patient moderate grade (2,3%)
Tuberous breast	Moderate	4 unilateral (included in UBH patient) (4,3%)	4 unilateral (included in UBH patient) (4,5%)
		12 bilateral (included in BBH patient moderate grade) (12,7%)	12 bilateral (included in BBH patient moderate grade) (13,4%)
Pseudoptosis	Low	4 (included in BBH patient low grade) (4,3%)	4 (included in BBH patient low grade) (4,5%)
Pectus excavatum	Moderate	5 (included in BBH patient moderate grade) (5,3%)	4 (included in BBH patient moderate grade) (4,5%)
	Low	3 (included in BBH patient moderate grade) (3,2%)	3 (included in BBH patient high grade) (3,4%)
Pectus carinatum	Low	0	4 (included in BBH patient moderate grade) (4,5%)

**Table 3:** Complicating factors classification

Deformities	BI group	LPF group
	(95 patients–SG)	(90 patients–CG)
Major complicating factors (M-CFs)	3 Vertical NAC asymmetry (3,2%)	3 Vertical NAC asymmetry -3%
	5 UBH(5,3%)	–
	–	23 BBH (high degree) (25,6%)
	–	12 Bilateral tuberous breast (13,4%)
	4 Unilateral tuberous breast(4,3%)	4 Unilateral tuberous breast (4,5%)
	5 Pectus excavatum (5,3%)	–
	0 Pectus carinatum 0%	–
	–	5 Low BMI (5,6%)
Minor complicating factors (m-CFs)	–	4 Pseudoptosis (4,5%)
	12 Bilateral tuberous breast (12,7%)	–
	10 Scoliosis (10,6%)	–
	4 Horizontal NAC asymmetries (4,3%)	4 Horizontal NAC asymmetries (4,5%)
	2 Areolar prolapse (2,2%)	2 Areolar prolapse (2,3%)
	5 Thin skins (5,3%)	–

### Statistical Analysis

A comparison between the SG and CG groups was conducted using the Student**’**s t-test or the Mann–Whitney test for responses to the self-assessment questionnaire. Data were expressed as mean (range), median (range), and percentages. A two-tailed p-value of less than 0.05 was considered statistically significant. An online *p* value calculator (https://www.graphpad.com/quickcalcs/ttest1.cfm) was used for all t-test analyses.

## Results

### Clinical Assessment

Augmentation mammoplasty using BI and LPF was successfully performed in all patients (both the SG and CG). Follow-up was completed for all patients (SG and CG) until the fourth year after the last procedure. However, several patients were unavailable for follow-up at T8—36 months and T9—48 months. Specifically, 44 patients (46.4%) in the SG and 52 patients (57,8%) in the CG were evaluated during the third year (T8), while 19 patients (20%) in the SG and 22 patients (24,5%) in the CG were evaluated during the fourth year (T9). The mean follow-up duration was 48 months (range: 12–60 months). Follow-up data at T6 and T8 in terms of LPF maintenance and breast surveillance were considered significant and showed (Table 1). At T6 (1-year post-procedure), 87,5% (n = 83) of patients treated with BI (SG) exhibited excellent cosmetic results (Fig. 1D-F), compared to 70% (n = 63) of patients in the CG. Breast augmentation maintenance and contour restoration in the SG were significantly higher than in the CG (*p* < .0001). However, more natural results were reported in the CG than in the SG (*p* < .0001).

In 76,8% (n = 69) of patients treated with LPF (CG), excellent cosmetic results were observed, including restoration of breast contour and an increase in three-dimensional volume by 40.5mm at T2 (3 weeks post-procedure), 24.5mm at T5 (6 months), and 22.5mm at T6 (12 months). The CG patients who underwent two procedures based on LPF achieved results comparable to those of BI at 1 year (T6), with an increase in the three-dimensional volume of 69.5mm (Fig. 2D-F), demonstrating cosmetic results like those obtained with definitive implants.

All patients in both the SG and CG expressed satisfaction with the resulting texture, softness, and volume contours. A majority in both groups were satisfied with the results (*p* = 0.412), indicated they would be willing to undergo the procedure again (*p* > 0.621) and would recommend the treatment to a friend (*p* = 0.333) (Table 4). Regarding self-evaluation of cosmetic outcomes after 1 year, scores ranged from 3 to 6 in the CG and from 1 to 4 in the SG (*p* = 0.096). The results suggest a strong trend of higher satisfaction among SG patients compared to CG patients (Table 4). Satisfaction-grade assessment via questionnaire revealed that all participants in both groups would choose to undergo breast augmentation with LPF or BIs, and they were adequately informed about the risks and complications of the treatments (including the risk of LPF resorption and the potential need for multiple treatments in the CG, as well as the risks of BI displacement and rejection in the SG).

**Table 4:** Patient’s satisfaction data

	BI group	LPF group
Patients no	95	90
Self-evaluation of cosmetic results (score range 1-6 / excellent–very poor)	83 (Fully satisfied) (87,4%):	63 (Fully satisfied) (70%)
	49 (Excellent/1) (51,6%)	40 (Excellent/1) (44,5%)
	26 (Very good/2) (27,4%)	20 (Very good/2) (22,3%)
	8 (Good/3) (8,5%)	3 (Good/3) (3,4%)
	6 (Not/fully/satisfied) (6,4%):	20 (Not/fully/satisfied) (22,3%)
	6 (Sufficient/4) (6,4%)	20 (Sufficient/4) (22,3%)
	6 (Not/satisfied) (6,4%):	7 (Not/satisfied) (7,8%)
	4 (Poor/5) (4,3%)	4 (Poor/5) (4,5%)
	2 (Very poor/6) (2,2%)	3 (Very poor/6) (3,4%)
Satisfaction of final result	89	83
	(93,7%)	(92,3%)
Recommend the treatment to a friend	89	83
	(93,7%)	(92,3%)
Available to breast augmentation	89	83
	(93,7%)	(92,3%)
Sufficiently informed about risks and complications	95	90
	−100%	−100%

When satisfaction was evaluated using a visual analog scale (VAS), both the SG and CG were similarly satisfied (*p* = 0.32). Figures 1 and 2 illustrate females categorized as showing “improvement” by all peers. When the new scores were calculated, patients in the SG and CG had average scores of 4.8 and 2.7, respectively (*p* = 0.31), indicating better overall improvement in the SG.

### Operative Outcomes: Instrumental Appraisal, Side Effects, LPF Maintenance, and Breast Surveillance

The mean number of sessions required for LPF was two. One session (mean transfer volume: 180 mL for breast, range 80-280 mL) was sufficient in 50 cases (56%). A second session (mean transfer volume: 210mL for breast, range 130-290mL) was necessary in the remaining 40 cases (44,5%), aiming to improve the breast volume and to have more similar results to the SG. CG patients treated with only one LPF showed 68,8% fat volume maintenance at T6 and 53% at T8 (3 years after the procedure), as documented by MRI. Fat necrosis was not identified. The only complication that occurred in the CG was the formation of oil cysts in 14 cases (16%). No second-look procedure was performed for side effects.

The mean number of interventions for BI was one. One session (mean implant volume: 235 mL for breast, range 150-320 mL) was sufficient in 86 cases (90%); 92 patients were treated with smooth matte and round BI, while 3 patients received texturized anatomic implants. A second-look procedure for side effects was performed in 9 cases (9%) (mean implant volume: 285ml), which included mastopexy for breast ptosis (n = 1), implant replacement for inadequate final volume (n = 2), and surgery for capsular contracture (n = 6).

Capsular contracture was identified in 6,4% of SG patients (n = 6) through ultrasound and MRI, while wrinkling was identified in 2 SG patients (2,2%). Rippling, intra- or extracapsular implant ruptures, and displacement were not observed. During the follow-up, side effects like NAC necrosis, skin necrosis, infections, and cancer were not detected in SG and CG.

### Complicating Factors Analysis

Chest deformities and breast deformities were identified in 53% of SG participants (n = 50) (17 patients with M-CFs, and 28 with m-CF) and 63,4% CG participants (n = 57) (47 patients with M-CFs, and 10 with m-CF). 22,3% of CG patients (n = 20) had “suboptimal results” compared with only 6,4% of SG patients (n = 6). The deformities had a greater detrimental impact on the CG findings than the SG. (DCC = 0.038, *p* <.001).

### Study Limitations and Strengths

The two most significant limitations were a) the design of the study as "open label" and b) the absence of Breast-Q-Scale evaluation. The ‘‘open label’’ trial, instead of ‘‘single blinded’’ or ‘‘double blinded,’’ prevents having an objective evaluation, or that, in any case, was not influenced in any way by the knowledge of having undergone a treatment rather than another. This constitutes a study bias. The Breast-Q scale was not used here because it was specific only to the BI group and not applicable to the LPF group (due to several irrelevant questions for LPF). Additionally, in each case, the bias was limited by the ‘‘custom-made approach’’ for every patient, both for SG and CG. Respectively, the size of the BI to use and the amount of LPF to inject were chosen based on the kind of defect. Additionally, the small number of patients who received anatomical implants (n = 3) may have limited the validity of conclusions regarding the natural appearance. A larger sample using an anatomical implant could potentially yield different comparative results. The three most significant strengths were a) the long-term follow-up, b) the topic originality, and c) the patient randomization. The follow-up of over three years in this study is one of the longest available in the current literature. Currently, this study is the only randomized trial in the literature about breast augmentation (fat vs implants):(https://pubmed.ncbi.nlm.nih.gov/?term=breast+augmentation+%28fat+vs+implants%29+randomized+&sort=date&size=100).

## Discussion

The breast volume assessment formula plays a vital role in pre-operative planning. It allows plastic surgeons to quantify the existing breast volume and determine the target volume based on patient goals and anatomical limitations. This information is essential not only for surgical symmetry and aesthetic outcomes, but also for deciding which technique (LPF *vs.* BIs) is most appropriate for a specific patient. Starting with this concept, the question could be: Is the outcome of LPF cases influenced by breast volume assessment? Yes, it is highly influenced. LPF is volume dependent, and the total achievable augmentation in a single session is often limited. If the initial breast volume is small and the desired increase is significant, multiple LPF sessions may be required, or it may even make implants a more feasible option. Conversely, for moderate volume increases or subtle contouring, LPF may provide a more natural outcome with fewer risks of complications such as capsular contracture or implant failure. In summary, integrating breast volume assessment into the decision-making process is essential to set realistic expectations, choose the optimal technique, and ultimately improve surgical outcomes. Further research into standardized volume calculation and predictive models could greatly enhance the personalization and efficacy of breast augmentation procedures.

Several analogies and differences were found between LPF and Bis procedures, producing advantages and disadvantages for each one, especially when complicating factors were detected. Breast augmentation with BIs typically takes between 1 and 2 hours, while it takes between 3 and 4 hours when it is performed with LPF. The recovery is usually rapid in both cases (BIs and LPF), with patients returning to light activities within two weeks. Full recovery and return to regular activities may take up to 6 weeks.

Effective surgical planning and patient education are crucial factors in achieving optimal outcomes in augmentation mammoplasty. Deformities of the breast or chest wall can make the procedure more complex and affect the final cosmetic result. Therefore, these deformities must be carefully considered during pre-operative assessments. Breast asymmetry is one of the most common deformities encountered in this context. Rohrich et al. [10] reported an incidence of 88% (n = 100), and DeLuca-Pytell et al. [11] found it to be 81.1% (n = 375).

In this randomized controlled trial, the author categorized complicating factors as either major or minor based on their impact on the surgery or the outcome. M-CFs were those that, when present alone, could lead to a suboptimal result. m-CFs had a less significant effect and could potentially go unnoticed if not corrected. However, when multiple (two or more) m-CFs were present, addressing these issues became more challenging. Additionally, m-CFs can easily be overlooked unless the pre-operative examination is thorough. Here, each M-CF was directly linked to suboptimal results. Furthermore, a combination of two or more m-CFs could lead to suboptimal outcomes.

It is important to note that suboptimal results in this study were directly based on patient satisfaction. The complicating factors are listed in Table 3, although additional major or minor factors, such as kyphosis or sunken chest, may exist in other surgeons**’** practices. The relationship between BMI and breast augmentation remains under-researched in the literature. Some studies suggest that both high and low BMI can lead to more post-operative complications. In this study, patients with a low BMI exhibited a diminished skin envelope, presenting challenges in BI and LPF treatments. Pectus excavatum is another deformity that can negatively affect aesthetic outcomes, especially when BI is used. A suitable option to camouflage this deformity is LPF. Subtle scoliosis deformities may also contribute to breast asymmetry [12]. There is a known correlation between the severity of scoliosis and breast volume differences [13]. The position of the NAC can be asymmetric both vertically and horizontally. Asymmetry in the horizontal plane was first described by Khan [14], who reported that 12% of patients exhibited NAC asymmetry in this axis. Augmentation mammoplasty (both in the case of BI and LPF) can lead to more pronounced lateralization of the NAC.

In the pre-operative phase, it is essential to evaluate breast volume by identifying areas that require correction, including an analysis of breast volume, shape, and symmetry. Kayar et al. [15] compared five methods for breast volume estimation: MG anthropometry, the Grossman–Roudner device, the Archimedes method, and the casting technique. Their study demonstrated that MG provided the most accurate results. Additionally, three-dimensional (3D) MRI evaluation has proved to be an effective and precise alternative for assessing breast volume, offering detailed insights into the in vivo shape and symmetry of the breast. MRI can accurately detect deformities, asymmetries, post-operative changes, and LPF volume loss. Furthermore, breast volume modifications and shape changes can be monitored during follow-up, thanks to the reproducibility of MRI assessments. [15, 16]

Regarding breast volume maintenance outcomes, 87,5% of SG patients treated with BI achieved excellent cosmetic results after one year, compared to 76,8% of patients in the control group (CG). The SG showed superior breast augmentation maintenance and contour restoration compared to the CG (*p* < 0.0001). CG patients who underwent two procedures based on LPF achieved results comparable to those of BI at 1 year (T6), with an increase in the three-dimensional volume of 69.5mm, demonstrating cosmetic results like those obtained with BIs. Conversely, the CG had higher rates of more natural results than the SG (*p* < 0.0001). When patient satisfaction was measured using a Visual Analog Scale (VAS), there was no significant difference between the SG and CG (*p* = 0.32). These results are consistent with the findings reported by Brault et al. [17] in their 2017 study comparing BI and LPF for breast augmentation, and they also appear to be in line with those previously published by Gentile [18]. This randomized study also assessed satisfaction levels in both the SG and CG groups using the VAS scale. In the SG group, the key advantages were long-term breast volume maintenance and the potential for a permanent result with a single procedure. In contrast, the CG group emphasized the more natural results and the absence of the need for a second treatment to address side effects. Additionally, the lack of an inframammary fold/peri-areolar scar in the CG group was crucial to the outcome. As expected, the CG group showed higher satisfaction with the appearance of the scar.

Despite the more noticeable and long-lasting results provided by the BIs and the appeal of the LPF technique for its natural outcome, certain issues persist with both methods. Specifically, for BIs, the use of a foreign object like a prosthesis raises concerns, including possible rejection or displacement or B-cell lymphoma. For LPF, challenges include maintaining the final breast volume, standardizing the injection technique, and the need for repeat treatments in some cases.

To date, only two studies (not randomized)—respectively, published by Brault et al. [17] and Gentile P [18].—have compared the results of BI and LPF in the treatment of tuberous breasts and breast hypoplasia.

The inclusion of M-CFs and m-CFs introduced variability that can obscure the core comparison between BI and LPF and could influence the choice of treatment. In these more complex cases, BI may provide more predictable volume and shape correction, whereas LPF may be limited due to fat resorption or insufficient volume gain.

## Conclusions

M-CFs and m-CFs in breast augmentation should be identified during the pre-operative assessment to recognize challenging cases and plan the most appropriate surgical approach. Issues such as breast deformities (including volume asymmetry, tuberous breasts, high-grade BBH, UBH, and NAC asymmetry), chest wall deformities (like pectus excavatum and carinatum), and very thin patients can negatively impact cosmetic outcomes and are considered M-CFs. The LPF technique yielded more natural results, providing good outcomes in patients with pectus excavatum, pectus carinatum, volume asymmetry, and UBH compared to BIs. On the other hand, two procedures based on LPF are necessary to have a similar BI outcome. BI procedures generally offered more noticeable and lasting results across most patients. On this basis, breast implants perform better in the presence of complicating factors.

More prospective and randomized studies are warranted soon to further evaluate and understand post-operative outcomes and patient satisfaction.

## Supplementary Information

Below is the link to the electronic supplementary material.

Supplementary file1 (DOCX 39 kb)

Supplementary file2 (DOCX 26 kb)

## References

[1] Bayram Y, Zor F, Karagoz H, et al. Challenging breast augmentations: the influence of preoperative anatomical features on the final result. Aesthet Surg J. 2016;36(3):313–20. 26420774 10.1093/asj/sjv181 PMC5127479

[2] Gentile P, Kothari A, Casella D, et al. Fat graft enhanced with adipose-derived stem cells in aesthetic breast augmentation: clinical, histological, and instrumental evaluation. Aesthet Surg J. 2020;40(9):962–77. 31637416 10.1093/asj/sjz292

[3] Gimble J, Guilak F. Adipose-derived adult stem cells: isolation, characterization, and differentiation potential. Cytotherapy. 2003;5:362–9. 14578098 10.1080/14653240310003026

[4] Gentile P, Scioli MG, Orlandi A, et al. Breast reconstruction with enhanced stromal vascular fraction fat grafting: what is the best method? Plast Reconstr Surg Glob Open. 2015;8(3):e406. 10.1097/GOX.0000000000000285 PMC4494476 26180707

[5] Araco A, Gravante G, Araco F, et al. Breast asymmetries: a brief review and our experience. Aesthet Surg J. 2006;30(3):309–19. 10.1007/s00266-005-0178-x 16733775

[6] Schuklenk U, Ashcroft R. International research ethics. Bioethics. 2000;14:158–72. 11765763 10.1111/1467-8519.00187

[7] Von Elm E, Altman DG, Egger M, et al. The strengthening the reporting of observational studies in epidemiology (STROBE) statement: guidelines for reporting observational studies. J Clin Epidemiol. 2008;61:344–9. 18313558 10.1016/j.jclinepi.2007.11.008

[8] Gentile P, De Angelis B, Di Pietro V, et al. Gentle is better: the original “gentle technique” for fat placement in breast lipofilling. J Cutan Aesthet Surg. 2018;11(3):120–6. 30533985 10.4103/JCAS.JCAS_24_18 PMC6243823

[9] Petit JY, De Lorenzi F, Rietjens M, et al. Technical tricks to improve the cosmetic results of breast-conserving treatment. Breast. 2007;16:13–6. 17070051 10.1016/j.breast.2006.08.004

[10] Rohrich RJ, Hartley W, Brown S. Incidence of breast and chest wall asymmetry in breast augmentation: a retrospective analysis of 100 patients. Plast Reconstr Surg. 2003;111(4):1513–9. 12618613 10.1097/01.PRS.0000049636.17522.1B

[11] DeLuca-Pytell DM, Piazza RC, Holding JC, et al. The incidence of tuberous breast deformity in asymmetric and symmetric mammaplasty patients. Plast Reconstr Surg. 2005;116(7):1894–9. 16327600 10.1097/01.prs.0000189206.94466.a9

[12] Fredricks S. Skeletal and postural relations in augmentation mammaplasty. Ann Plast Surg. 1978;1(1):44–7. 727652 10.1097/00000637-197801000-00010

[13] Tsai FC, Hsieh MS, Liao CK, et al. Correlation between scoliosis and breast asymmetries in women undergoing augmentation mammaplasty. Aesthet Plast Surg. 2010;34(3):374–80. 10.1007/s00266-010-9506-x 20383498

[14] Khan UD. Breast augmentation in asymmetrically placed nipple-areola complex in the horizontal axis: lateralization of implant pocket to offset lateralised nipples. Aesthet Plast Surg. 2009;33(4):591–6. 10.1007/s00266-009-9324-1 19296149

[15] Kayar R, Civelek S, Cobanoglu M, et al. Five methods of breast volume measurement: a comparative study of measurements of specimen volume in 30 mastectomy cases. Breast Cancer. 2011;5:43–52. 21494401 10.4137/BCBCR.S6128 PMC3076010

[16] Costantini M, Cipriani A, Belli P, et al. Radiological findings in mammary autologous fat injections: a multi-technique evaluation. Clin Radiol. 2013;68:27–33. 22749812 10.1016/j.crad.2012.05.009

[17] Brault N, Stivala A, Guillier D, et al. Correction of tuberous breast deformity: a retrospective study comparing lipofilling versus breast implant augmentation. J Plast Reconstr Aesthet Surg. 2017;70(5):585–95. 28341593 10.1016/j.bjps.2017.02.011

[18] Gentile P. Breast silicone gel implants versus autologous fat grafting: biomaterials and bioactive materials in comparison. J Clin Med. 2021;10(15):3310. 34362094 10.3390/jcm10153310 PMC8348805

