# Supplementary material for: Breast Implants or Lipofilling in Augmentation Mammoplasty? A Randomized, Open-Label, Controlled Trial
Source: Aesthetic Plast Surg. 2025 Sep 4;50(3):1044–56. doi: 10.1007/s00266-025-05204-0 (PMC12992393; doi:10.1007/s00266-025-05204-0)
Supplement: Supplementary file 1 — Supplementary file1 (DOCX 39 kb) [file 266_2025_5204_MOESM1_ESM.docx]

**STROBE Statement—checklist of items that should be included in reports of observational studies.**

|  | Item No. | Recommendation | Page  No. | Relevant text from manuscript |
| --- | --- | --- | --- | --- |
| **Title and abstract** | 1 | **Title:** Breast implants or lipofilling in augmentation mammoplasty? A randomized, open-label, controlled trial. |  |  |
|  |  | **ABSTRACT**  **Background:** Augmentation mammoplasty strategies have evolved over the last twenty-five years. Breast silicone implants and fat grafting are used individually or combined in a hybrid technique. The author presents his own experience using breast implants (BI) or fat grafting, commonly called lipofilling (LPF), to correct breast hypoplasia.  **Objectives:** Compare the aesthetic results obtained in a study group (SG) using BI in breast hypoplasia correction with those of a control group (CG) treated with LPF, also analyzing the influence of breast and chest deformities (tuberous breast, breast volume differences/asymmetries, nipple-areola complex asymmetry, pectus excavatum, and carinatum).  **Methods:** A randomized, open-label controlled study was performed. 95 patients affected by breast hypoplasia (SG) were treated with BI, comparing results with the CG (n = 90) treated with LPF. The pre-operative analysis was conducted through an accurate anamnesis (considering also the patient's expectative), clinical and photographic assessment, and an instrumental evaluation based on magnetic resonance imaging, mammography, and ultrasound. Post-operative follow-up occurred at 1, 2, 4, weeks, 3, 6, 12 months, and then annually until the fourth year.  **Results:** 87,5% (n = 83) of SG patients treated with BI showed excellent aesthetic outcomes after 12 months compared with the CG patients treated with LPF, who showed the same results in 70% (n = 63) of cases. Breast augmentation maintenance and contour restoration in the SG were significantly higher than in the CG (p < .0001). However, more natural results were reported in the CG than in the SG (p < .0001).  **Conclusions:** BI and LPF were safe and effective in this controlled trial. CG's patients displayed more natural results, additionally obtaining a better pectus excavatum correction, while SG's patients showed more evident and lasting results. |  |  |
| Introduction | | | |  |
| Background/rationale | 2 | Augmentation mammoplasty strategies have evolved over the last twenty-five years. Breast silicone implants and fat grafting are used individually or combined in a hybrid technique. The author presents his own experience using breast implants (BI) or fat grafting, commonly called lipofilling (LPF), to correct breast hypoplasia. |  |  |
| Objectives | 3 | This article aims to compare the aesthetic results obtained in a study group (SG) using BI in breast hypoplasia correction with those of a control group (CG) treated with LPF, also analyzing the influence of breast and chest deformities (tuberous breast, breast volume differences/asymmetries, nipple-areola complex asymmetry, pectus excavatum, and carinatum). |  |  |
| Methods | | | |  |
| Study design | 4 | A randomized, open-label, controlled trial, classified as evidence-based medicine (EBM) level 1, was performed fully respecting the Declaration of Helsinki and internationally consented ethics in clinical research. |  |  |
| Setting | 5 | Quality assessments were performed on the following criteria:  – Surveys related to the patient’s grade of satisfaction on resulting breast size, breast shape, breast lift, breast and NAC symmetry, scar quality, sexual well-being, availability to undergo the procedure again, to recommend the treatment to friends, and sufficient information about the randomized trial, risks and side effects (range vote 4 to 9: Excellent [9]; extremely satisfied [8]; satisfied [7]; neutral [6]; dissatisfied [5]; very dissatisfied [4]) (Appendix A);  – Clinical evaluation using the physician's overall assessment score (excellent [9]; good [8]; discreet [7]; enough [6]; poor [5]; inadequate [4]);  – Clinical evaluation using the patient’s overall assessment score (from excellent to very dissatisfied);  – Visual Analog Scale (VAS) (range 1 to 10);  – Additional Factors/variables, such as breast asymmetry, breast hypoplasia, NAC asymmetries, pseudoptosis, glandular ptosis, tuberous breast and areolar prolapse, and chest deformities such as pectus excavatum and carinatum;  – Side effects signaling (presence or absence). |  |  |
| Participants | 6 |  |  |  |
|  |  | Randomized controlled open-label study. 185 patients were analyzed (95 study group and 90 control group). Inclusion and exclusion criteria were considered during the enrolment. |  |  |
| Variables | 7 | The two most significant limitations were a) the design of the study as "open-label" and b) the absence of Breast-Q-Scale evaluation. The ‘‘open-label’’ trial, instead of ‘‘single-blinded’’ or ‘‘double-blinded,’’ prevents having an objective evaluation, or that, in any case, was not influenced in any way by the knowledge of having undergone a treatment rather than another. This constitutes a study bias. The Breast-Q scale was not used here because it was specific only to the BI group and not applicable to the LPF group (due to several irrelevant questions for LPF). Additionally, in each case, the bias was limited by the ‘‘custom-made approach’’ for every patient, both for SG and CG. Respectively, the size of the BI to use and the amount of LPF to inject were chosen based on the kind of defect. |  |  |
| Data sources/ measurement | 8* | Breast size, breast shape, breast lift, breast and NAC symmetry, scar quality, sexual well-being, availability to undergo the procedure again, to recommend the treatment to friends, and sufficient information about the randomized trial, risks and side effects |  |  |
| Bias | 9 | The "open-label" study, as opposed to "single-blinded" or "double-blinded," inhibits having an entirely impartial evaluation, or that in any case was not impacted in any way by knowledge of having received one therapy rather than another. This entails a bias in the research. |  |  |
| Study size | 10 | 185 patients analyzed |  |  |

Continued on next page

| Quantitative variables | 11 | None |  |  |
| --- | --- | --- | --- | --- |
| Statistical methods | 12 | A comparison between SG and CG was done with the student’s 𝑡-test or Mann-Whitney for the question of the self-assessment questionnaire. The data is expressed by mean (range), median (range), and percentages. A two-tailed p-value less than 0.05 has been identified as significant. All t-test analyses were performed using an online *p*-value calculator (<https://www.graphpad.com/quickcalcs/ttest1.cfm>). |  |  |
| Results | | | | |
| Participants | 13* | Augmentation mammoplasty using BI and LPF was successfully performed in all patients (both the SG and CG). Follow-up was completed for all patients (SG and CG) until the fourth year after the last procedure. However, several patients were unavailable for follow-up at T8 – 36 months and T9 – 48 months. Specifically, 44 patients (46.4%) in the SG and 52 patients (57,8%) in the CG were evaluated during the third year (T8), while 19 patients (20%) in the SG and 22 patients (24,5%) in the CG were evaluated during the fourth year (T9). The mean follow-up duration was 48 months (range: 12–60 months). Follow-up data at T6 and T8 in terms of LPF maintenance and breast surveillance were considered significant and showed (Table 1). At T6 (1-year post-procedure), 87,5% (n = 83) of patients treated with BI (SG) exhibited excellent cosmetic results, compared to 70% (n = 63) of patients in the CG. Breast augmentation maintenance and contour restoration in the SG were significantly higher than in the CG (p < .0001). However, more natural results were reported in the CG than in the SG (p < .0001).  In 76,8% (n = 69) of patients treated with LPF (CG), excellent cosmetic results were observed, including restoration of breast contour and an increase in three-dimensional volume by 43.3mm at T2 (3 weeks post-procedure), 29.5mm at T5 (6 months), and 25.7mm at T6 (12 months). The CG patients who underwent two procedures based on LPF achieved results comparable to those of BI at 1 year (T6), with an increase in the three-dimensional volume of 72.8mm, demonstrating cosmetic results like those obtained with definitive implants. All patients in both the SG and CG expressed satisfaction with the resulting texture, softness, and volume contours. A majority in both groups were satisfied with the results (p = 0.412), indicated they would be willing to undergo the procedure again (p > 0.621), and would recommend the treatment to a friend (p = 0.333) (Table 4). Regarding self-evaluation of cosmetic outcomes after 1 year, scores ranged from 3 to 6 in the CG and from 1 to 4 in the SG (p = 0.096). The results suggest a strong trend of higher satisfaction among SG patients compared to CG patients (Table 4). Satisfaction-grade assessment via questionnaire revealed that all participants in both groups would choose to undergo breast augmentation with LPF or BIs, and they were adequately informed about the risks and complications of the treatments (including the risk of LPF resorption and the potential need for multiple treatments in the CG, as well as the risks of BI displacement and rejection in the SG. When satisfaction was evaluated using a visual analog scale (VAS), both the SG and CG were similarly satisfied (p = 0.32). Figure 4 illustrates females categorized as showing “improvement” by all peers. When the new scores were calculated, patients in the SG and CG had average scores of 4.8 and 2.7, respectively (p = 0.31), indicating better overall improvement in the SG. |  |  |
| Descriptive data | 14* | Additionally, the mean number of sessions required for LPF was two. One session (mean transfer volume: 180 mL for breast, range 80-280 mL) was sufficient in 50 cases (56%). A second session (mean transfer volume: 210mL for breast, range 130-290mL) was necessary in the remaining 40 cases (44,5%) aiming to improve the breast volume and to have more similar results to the SG. CG patients treated with only one LPF showed 68,8% fat volume maintenance at T6 and 53% at T8 (3 years after the procedure), as documented by MRI. Fat necrosis was not identified. The only complication that occurred in the CG was the formation of oil cysts in 14 cases (16%). No second-look procedure was performed for side effects.  The mean number of interventions for BI was one. One session (mean implant volume: 235 mL for breast, range 150-320 mL) was sufficient in 86 cases (90%). 92 patients were treated with smooth matte and round BI, while 3 patients received texturized anatomic implants. A second-look procedure for side effects was performed in 9 cases (9%), (mean implant volume: 285ml), which included mastopexy for breast ptosis (n = 1), implant replacement for inadequate final volume (n = 2), and surgery for capsular contracture (n = 6).  Capsular contracture was identified in 6,4% of SG patients (n = 6) through ultrasound and MRI while wrinkling in 2 SG patients (2,2%). Rippling, intra- or extracapsular implant ruptures, and displacement were not observed. During the follow-up, side effects like NAC necrosis, skin necrosis, infections, and cancer were not detected in SG and CG. |  |  |

Continued on next page

| Other analyses | 17 | None |  |  |
| --- | --- | --- | --- | --- |
| Discussion | | | | |
| Key results | 18 | After a detailed analysis of the data here presented, breast hypoplasia with breast and/or chest deformities can be adequately corrected, in selected patients, by BIs and LPF. The advantages are the more natural results in LPF group and more stable results in BI group, while the disadvantages may be fat resorption and oil cysts in LPF group, while capsular contracture in BI group.. |  |  |
| Limitations | 19 | The two most significant limitations were a) the design of the study as "open-label" and b) the absence of Breast-Q-Scale evaluation. The ‘‘open-label’’ trial, instead of ‘‘single-blinded’’ or ‘‘double-blinded,’’ prevents having an objective evaluation, or that, in any case, was not influenced in any way by the knowledge of having undergone a treatment rather than another. This constitutes a study bias. The Breast-Q scale was not used here because it was specific only to the BI group and not applicable to the LPF group (due to several irrelevant questions for LPF). Additionally, in each case, the bias was limited by the ‘‘custom-made approach’’ for every patient, both for SG and CG. Respectively, the size of the BI to use and the amount of LPF to inject were chosen based on the kind of defect. |  |  |
| Interpretation | 20 | The procedure tested appeared to be effective in the treatment of breast hypoplasia |  |  |
| Generalisability | 21 | Not applicable |  |  |
| Other information | |  | | |
| Funding | 22 | The study protocol has been developed in agreement with research contract #1467/2017 and associate professor contract #13489/2021 between the author and the University of Rome “Tor Vergata”, Italy, and as a part of a research project approved by the Surgical Science Department with number: E83C22001960005. |  |  |

Information on the STROBE Initiative is available at www.strobe-statement.org.
