# Supplementary material for: Breast Implants or Lipofilling in Augmentation Mammoplasty? A Randomized, Open-Label, Controlled Trial
Source: Aesthetic Plast Surg. 2025 Sep 4;50(3):1044–56. doi: 10.1007/s00266-025-05204-0 (PMC12992393; doi:10.1007/s00266-025-05204-0)
Supplement: Supplementary file 2 — Supplementary file2 (DOCX 26 kb) [file 266_2025_5204_MOESM2_ESM.docx]

**Appendix 1. Patient’s satisfaction questionnaire.**

Degree of Satisfaction:

9 - Excellent;

8 – Extremely satisfied;

7 – Satisfied;

6 – Neutral;

5 – Dissatisfied;

4 – Very dissatisfied.

In the pre-operative period

According to the scale above, grade questions 1 through 6 according to your degree of satisfaction:

1. How do you feel about your breast size?

2. How do you feel about your breast shape?

3. How do you feel about your breast and nipple-areola complex (NAC) symmetry?

4. How do you feel about your breast ptosis?

5. How comfortable feel during sexual activity?

6. If you have already undergone mammoplasty surgery, how do you feel about your scar quality?

7. Did you have any pieces of information on risk and complications (including the risk of NAC asymmetry, residual chest or breast deformities, bad scars, implant displacement or rejection, fat resorption or displacement, and the possibility of repeating the treatment)?

8. Are you aware that you are part of a randomized trial in which the surgical technique may be different from the traditional one, but still suitable for correcting your defect?

9. Based on all the information received in the pre-surgery, would you recommend the same route to friends?

10. Are you available to undergo this procedure?

In the postoperative period at 1 week (T1), 2 weeks (T2), 4 weeks (T3), 3 months (T4), 6 months (T5), 12 months (T6), and then annually.

According to the scale above, grade questions 1 through 6 according to your degree of satisfaction:

1. How do you feel about your breast size?

2. How do you feel about your breast shape?

3. How do you feel about your breast and nipple-areola complex (NAC) symmetry?

4. How do you feel about your breast lift?

5. How comfortable feel during sexual activity?

6. How do you feel about the quality of your peri-areolar scars?

7. Did you have any pieces of information on risk and complications (including the risk of NAC asymmetry, residual chest or breast deformities, bad scars, implant displacement or rejection, fat resorption or displacement, and the possibility of repeating the treatment)?

8. Were you informed in detail that you were part of a randomized study in which the operative technique, (however, indicated for the correction of your defect), was chosen randomly?

9. Based on your post-op, do you recommend the treatment to friends?

10. Are you available to undergo this procedure again?
